# Detection and genetic diversity of subgroup K avian leukosis virus in local chicken breeds in Jiangxi from 2021 to 2023

**DOI:** 10.3389/fmicb.2024.1341201

**Published:** 2024-02-08

**Authors:** Fanfan Zhang, Haiqin Li, Cui Lin, Yue Wei, Weihong Zhang, Yanping Wu, Zhaofeng Kang

**Affiliations:** Institute of Animal Husbandry and Veterinary Medicine, Jiangxi Academy of Agricultural Sciences, Nanchang, China

**Keywords:** avian leukosis virus subgroup K, prevalence, genetic diversity, gp85 gene, avian leukosis virus

## Abstract

Avian leukosis virus subgroup K (ALV-K) is a new subgroup of avian leukosis virus (ALV) that was first identified in Chinese native chickens in recent years. To further understand the molecular epidemiology and evolutionary diversity of ALV-K, this study investigated the molecular epidemiology of 73,664 chicken plasma samples collected from Jiangxi native chicken flocks. The results showed that ALV-J was the most predominant ALV subtype in Jiangxi native chickens, with a high positivity rate of 4.34%. From 2021 to 2023, there was a gradual upward trend in the proportion of positive numbers of ALV-K among ALV-positive samples, and there was a trend of outbreaks. ALV-J and ALV-K were the main co-infection patterns. Genetic evolutionary analysis based on ALV-K gp85 gene showed that the isolated ALV-K in this study were distributed in various branches of the evolutionary tree with genetic diversity. The homology results showed that the amino acid homology of the isolated ALV-K gp85 gene ranged from 33.9 to 88.1% with the reference strains of subtypes A, B, C, D, E, and J, and from 91.9 to 100% with the other ALV-K reference strains. Multiple mutations were present in the ALV-K gp85, and especially significant mutations were found in the highly variable region hr2. The results of ALV-K replication efficiency showed that the replication efficiency of ALV-K was significantly lower than that of ALV-J. These results enriched the genome sequence data of ALV-K in Chinese geoducks, and laid the foundation for further research on the pathogenesis and prevention of ALV-K.

## 1 Introduction

Avian leukosis (AL) is an avian neoplastic disease caused by avian leukosis virus (ALV) infection of the avian C retrovirus group, including lymphoblastic leukosis, myeloid leukosis and erythrocytic leukosis (Deng et al., 2022; Mo et al., 2022). According to the host range and the mutual interference between different strains and the antigenic structure of the viral capsular glycoprotein, ALV can be divided into 11 subgroups (A to K), of which subgroup E belongs to the endogenous ALV, which is not pathogenic or has very low pathogenicity, while subgroup K of ALV (ALV-K) is a new subgroup firstly isolated from the local strains of chickens in our country, and has been reported more frequently in yellow-feathered broilers and local strains of chickens in recent years (Lv et al., 2019; Chen et al., 2022; Guo et al., 2023). More. Compared with other subgroups, subgroup J has stronger pathogenicity and transmission ability, and is more harmful (Smith et al., 2018; Deng et al., 2021). Epidemiological investigation showed that A, B, J, and K are the four major subgroups with the widest epidemiological scope and the most serious harm in China (Shao et al., 2017). It is worth noting that although ALV-K has been prevalent in China for more than a decade, there is still no long-term systematic epidemiological study on Jiangxi local breed chickens. Different breeds of chickens infected with ALV exhibit different types of clinical symptoms, such as growth retardation, low feed utilization, multiple organ tumors and induced immunosuppression, causing serious economic losses to the poultry farming industry.

In 2012, Wang et al. isolated a novel strain of ALV from local breed chickens in Jiangsu province, whose env gene differed significantly from other subgroups, and it was considered a new subgroup of avian leukosis virus and named ALV-K (Li et al., 2016; Guo et al., 2023). In previous studies, an exogenous ALV strain TW-3593 isolated from Taiwan, China, in 2008, and several strains of fowl glioma virus (FGV) isolated from Japan were reported to be from local chickens, which showed high amino acid sequence similarity (more than 90%) to the gp85 gene of Chinese ALV-K isolates, but low similarity to other ALVs (Chang et al., 2013; Guo et al., 2023). Subsequently, ALV-K isolates were also found in yellow chickens from Guangdong and Shandong, China (Li et al., 2016, 2019; Sun et al., 2020). Recently, our research group found that ALV-K is widespread in endemic chickens in Jiangxi Province, southern China, and the positivity rate in ALV-positive samples has been increasing yearly.

Currently, there is no vaccine or effective drug for the control of ALV-K-induced diseases. Eradication of infected populations is considered the most effective way to minimize the spread of ALV-K in developed poultry industries; however, control and eradication of ALV-K in less organized poultry industries such as local breeds of chickens, free-range chickens and hobby chickens is still a major challenge (Su et al., 2018a; Li et al., 2023). It has been frequently reported in local breed chickens in China in the last decade and must be monitored and studied to avoid it as a potential risk. For this reason, the present study was conducted to investigate the epidemiology of the four most damaging avian leukosis viruses in local chicken breeds in Jiangxi (Taihe Silky Fowl, Dongxiang Green-Shell Chicken, and Nindu yellow chickens), and to perform whole-genome sequencing, sequence comparisons, and genetic evolutionary analyses of isolated ALV-K representative strains, with a view to provide a basis for epidemiological investigations of ALV in subgroup K in China.

## 2 Materials and methods

### 2.1 Animal ethics statement

The Experimental Animal Ethics Committee in Jiangxi Academy of Agricultural. Sciences (JXAAS 2020-0052) approved this study. All procedures involving animals were conducted according to the guidelines for the care and use of experimental animals established by the Ministry of Agriculture of China.

### 2.2 Clinical samples and cells

A total of 73,664 plasma samples were collected during 2022–2023 from Taihe Silky Fowl, Dongxiang Green Shell Laying Chicken, Chongren Partridge Chicken and Ningdu Yellow Chicken groups, respectively, which were undergoing AL purification. The samples were collected from local chickens of different breed groups: 19,008 samples from Taihe Silky Fowl (29–30 weeks), 35,922 samples from Broken Dongxiang Green-Shell Chicken (27–28 weeks), 7685 samples from Raw Chongren Partridge Chicken (22–23 weeks), and 11,049 samples from Ningdu Yellow Chicken (20–21 weeks). Among them, the anticoagulated blood samples were collected aseptically and then centrifuged at 3,000 r/min for 5 min to obtain plasma homogenate, which was stored at −80°C. DF-1 cells (CRL-12203, ATCC), which can distinguish between endogenous and exogenous ALV, were purchased from ATCC (American Type Culture Collection), and the growth solution required for their culture was DMEM containing 10% fetal bovine serum (FBS, GIBCO, United States). The maintenance solution was DMEM containing 1% FBS. DF-1 cells were maintained in Dulbecco’s modified Eagle’s medium supplemented with 5% fetal bovine serum and were kept in our own laboratory.

### 2.3 Virus isolation

Each chicken was aseptically collected 1 mL of anticoagulated blood through the wing vein with a sterile sodium heparin anticoagulant blood collection tube, centrifuged at 1,200 r/min for 3 min at 4°C, and then 60 uL of plasma samples were inoculated on DF-1 cells that had grown into monolayers in a 96-well cell culture plate, and then incubated at 37°C for 2 h. After discarding the cell growth solution, the cells were washed twice with sterile PBS and then replaced by the cell maintenance solution, and then cultured for 9 day under the condition of 37°C and 5% CO_2_. After incubation for 9d at 37°C and 5% CO_2_, the culture supernatant was collected and tested by ALV-p27 antigen ELISA kit (NECVB, China) according to the instructions to determine the presence of exogenous ALV infection, and the supernatant of p27-positive samples was stored at −80°C for spare use.

### 2.4 PCR amplification and identification of proviral genes

The collected cells and supernatant were lysed by adding DNAiso reagent, and then purified and lysed to obtain DNA, which was used as a template for PCR amplification of proviral genes. Four pairs of primers for the identification of ALV subgroups A, B, J, and K were synthesized by Sangon Biotech (Shanghai) Co., Ltd. The primer sequences are shown in Table 1. PCR amplification system (total volume of 25 μL): 1 μL each of upstream and downstream primers, 12.5 μL of 2 × Taq Master Mix (Dye Plus), 2 μL of DNA template, 8.5 μL of ddH_2_O. PCR amplification procedure: pre-denaturation at 95°C for 5 min, denaturation at 94°C for 30 s, annealing at 58°C for 45 s, and extension at 72°C for 60 s for 35 cycles. A blank control (normal DF1 cells) and a positive control [ALV-K prototype virus (TW-3593)] were set at the same time. PCR products were taken and identified by 1% agarose gel electrophoresis.

**Table 1:** Primers used in detection of ALV-A, ALV-B, ALV-J, ALV-K and amplifying the whole genome of ALV-K.

Primer	Sequence	Length (bp)	Purpose
ALV-K-1F	ATCGATTGTAGTCAAATAGAGCCAGAGGC	2,497	Whole genome
ALV-K-1R	AGGGGTGTCTAAGGAGAAACCG		
ALV-K-2F	ACCCGGAGAAGACACCCTT	2,672	
ALV-K-2R	CTGGGTCGGTCAGAAGGATGT		
ALV-K-3F	ACCCGGAGAAGACACCCTT	2,313	
ALV-K-3R	TATAGCGGAGGAGGAGCCACCTCGT		
ALV-F	ACCCGGAGAAGACACCCTT		Detection (Zhang et al., 2021)
ALV-A-R	AGGGGTGTCTAAGGAGAAACCG	563	
ALV-B-R	CTGGGTCGGTCAGAAGGATGT	563	
ALV-J-R	CATAGGGCCTTATAAGAAGGTCAT	563	
ALV-K-R	TATAGCGGAGGAGGAGCCACCTCGT	559	

### 2.5 Amplification and sequencing of the provirus genome

The proviral DNA was divided into three segments, and three pairs of overlapping primers specific for the whole ALV-K gene were synthesized according to the method in the reference (Table 1), and the proviral DNA was used as a template for PCR amplification. PCR amplification system (total volume of 25 μL): 1 μL each of upstream and downstream primers, 12.5 μL of 2 × Vazyme LAmp Master Mix (Dye Plus), 2 μL of DNA template, 8.5 μL of ddH_2_O. PCR amplification procedure: pre-denaturation at 95°C for 5 min, denaturation at 95°C for 30 s, annealing at 58°C for 30 s, extension at 72°C for 2 min, a total of 35 cycles; 72°C extension for another 10 min. PCR amplification products were identified and the target bands were cut off on 1.0% agarose gel electrophoresis and purified by commercial gel recovery reagents. The purified DNA was ligated into pMD18-T Vector (TaKaRa Co., Ltd., Dalian, China) and transformed into *E. coli* receptor cells DH5aα. Single colonies were extracted with Plasmid MiniKit (OMEGA, United States) and identified by enzyme digestion, and the positive cloned plasmids were sent to Shanghai Sangong Biological Engineering Co.

### 2.6 Phylogenetic analysis

Reference strains with different domestic and international ALV subtypes, years and regions, and host sources were selected from NCBI (Table S1). The full-length splicing of each fragment sequence of the isolates was performed with DNAStar 7.0/SeqMan software, and the full and partial gene sequences of each ALV-K strain were compared with BioEdit 7.2.6.1 software for nucleic acid and amino acid homology analysis; and the genetic evolutionary tree of the env gene of the isolates was constructed using Mega 7.0 software (using the Neighbor-Joining Neighbor-Joining (NJ) method with Bootstrap value of 1,000), to assess their origin and evolutionary trend. The whole gene and gp85 gene of the reference and isolate viruses were analyzed by the Clustal W method using DNAStar 7.0 software.

### 2.7 Analysis of replication ability through real-time quantitative PCR and 50% tissue culture infective dose

Briefly, 6-well cell culture plates containing monolayers of DF-1 cells were prepared prior to infection and labeled A, B and C (Li H. Q. et al., 2022). Plate A was inoculated with 10^5^ 50% tissue culture infectious dose (TCID_50_) of JXDX2301 per well, plate B was inoculated with 10^5^ TCID_50_ of JXTH2202 per well and plate C was inoculated with 10^5^ TCID_50_ of JXTH2203 per well. The titer of the virus was further determined from the collected cell supernatant at 1 to 7-day post-infection (dpi). The viral solution was multiply diluted at 10^1^ to 10^7^ with 8 replicates per dilution and inoculated onto the surface of DF-1 cells in 96-well plates. After incubation for 2 h, the viral solution was run off and replaced with 1% FBS in DMEM culture medium for maintenance for 7 dpi. The p27 antigen was determined by IFA, and the TCID_50_ value was calculated according to the Reed-Muench method in three independent replicates of the assay (for detailed methodology, see Wu et al., 2011).

## 3 Results

### 3.1 Virus detection and isolation

Infecting the samples with DF-1 cells produced no significant cytopathic effect (CPE) compared to the control. DF-1 cell supernatants were further tested using the ALV ELISA kit (NECVB, China), and a total of 5,684 samples were found to be positive out of 73,664 samples. PCR of the obtained positive samples using primers for different ALV subgroups (Table 2) showed that a total of 35 samples were positive for ALV-A, 13 samples were positive for ALV-B, 3,200 samples were positive for ALV-J, and 2,436 samples were positive for ALV-K.

**Table 2:** Categorization of detection results on Avian leukosis virus of samples collected from 2021 to 2023.

Classifications	Sample No.	Viruses [number (positive rate, %)]			
		ALV-A	ALV-B	ALV-J	ALV-K
**Year**					
2021	23,560	15 (0.06%)	5 (0.02%)	2001 (8.49%)	753 (3.20%)
2022	25,694	14 (0.05%)	3 (0.01%)	840 (3.27%)	941 (3.66%)
2023	24,410	6 (0.02%)	5 (0.02%)	359 (1.47%)	742 (3.04%)
Total	73,664	35 (0.05%)	13 (0.02%)	3,200 (4.34%)	2,436 (3.31%)
**Species**					
TSF	19,008	6 (0.03%)	2 (0.01%)	556 (2.93%)	507 (2.67%)
DGSC	35,922	26 (0.07%)	10 (0.03%)	2,410 (6.71%)	1745 (4.86%)
CPC	7,685	3 (0.04%)	1 (0.01%)	195 (2.54%)	128 (1.67%)
NYC	11,049	0 (0.00%)	0 (0.00%)	39 (0.35%)	56 (0.51%)
**Genders**					
Roosters	65,054	35 (0.05%)	13 (0.02%)	3,035 (4.67%)	2,329 (3.58%)
Hens	8,610	0 (0.00%)	0 (0.00%)	165 (1.92%)	107 (1.24%)

TSF, Taihe Silky Fowl; DGSC, Dongxiang Green-Shell Chicken; CPC, Chongren Partridge Chicken; NYC, Ningdu Yellow Chicken.

### 3.2 Prevalence of ALV-A, ALV-B, ALV-J, and ALV-K

**Figure 1:**
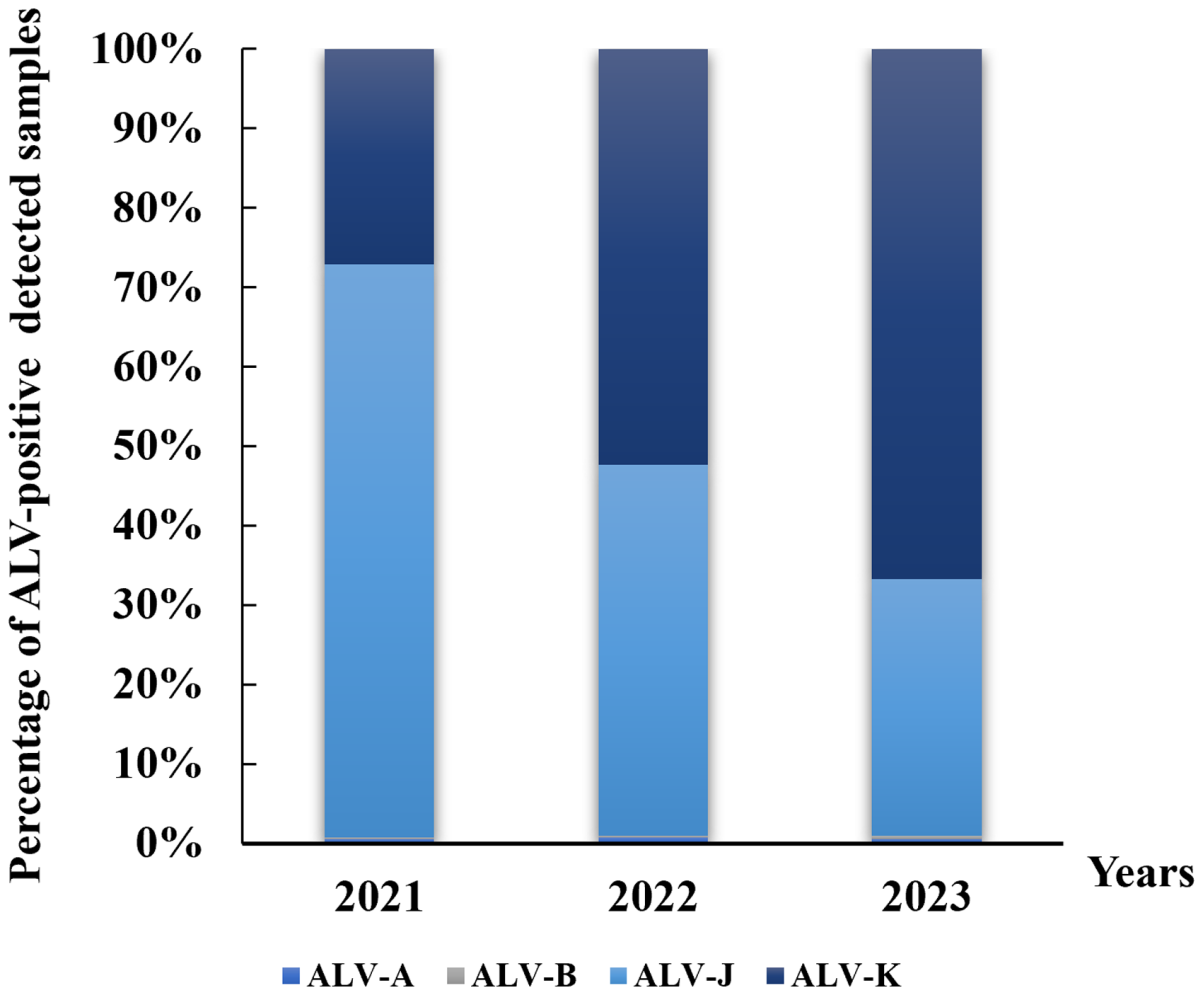
Distribution of avian leukemia virus in local breeders in Jiangxi, China, 2021–2023. ALV-A, avian leukosis virus subgroup A; ALV-B, avian leukosis virus subgroup B; ALV-J, avian leukosis virus subgroup J; ALV-K, avian leukosis virus subgroup K.

A total of 73,664 anticoagulated blood samples from four major local chicken breeds in Jiangxi from 2021 to 2023 were tested in this study. The results showed that ALV-J remained the predominant subtype of ALV in infected chickens, with prevalence rates ranging between 1.47 and 8.49% (Table 2). This was followed by subtype ALV-K with prevalence rates ranging from 3.04 to 3.66%. low detection rates (<0.06%) were observed for ALV-A and ALV-B. From the statistics of the percentage of each subtype in the ALV-positive samples, the positive rate of ALV-J subtype was decreasing year by year, while it is noteworthy that the positive rate of ALV-K was increasing year by year (Figure 1). Samples infected with four ALV subtypes were counted in the four sampled local chicken breeds. The highest ALV detection rate was found in DGSC, with ALV-A, ALV-B, ALV-J, and ALV-K positivity rates of 0.07, 0.3, 6.71, and 4.86%, respectively; the lowest ALV detection rate was found in NYC, with ALV-J and ALV-K positivity rates of 0.35 and 0.51%, respectively; and no ALV-A, ALV-B infection. In terms of the sex of the samples, the ALV positivity rate was significantly higher (*p* < 0.05) in hen flocks (8.32%) than in cockerel flocks (1.24%). These data suggest that ALV-J and ALV-K remain the predominant subtypes of ALV in flocks, especially in farms undergoing ALV evolutionary procedures, where ALV-K was significantly elevated.

### 3.3 Co-infections of ALV In chickens in Jiangxi

The frequency of co-infections was analyzed in this study. In 73,664 anticoagulated blood samples, the prevalence of mono-infection was 0.01% (9/73,664), 0.01% (9/73,664), 3.75% (2,762/73,664) and 2.68% (1,977/73,664) for ALV-A, ALV-B, ALV-J, and ALV-K, respectively (Table 3). The prevalence of co-infection caused by more than one ALV subtype ranged from 0.00 to 0.59%. The highest prevalence of co-infection with ALV-J and ALV-K was 0.59%. In addition, triple infections were observed in six cases, including three cases of co-infection with ALV-A, ALV-J, and ALV-K, and three cases of co-infection with ALV-B, ALV-J, and ALV-K.

**Table 3:** Mono-and co-infections of ALV-A, ALV-B, ALV-J, and ALV-K in samples during 2021 to 2023.

Mono-and co-infections of ALVs	Sample No.	Local chicken breeds resources of Jiangxi, China [Number (positive rate, %)]			
		TSF	DGSC	CPC	NYC
Only A	73,664	1 (0.00)	6 (0.01)	2 (0.00)	0 (0.00)
Only B	73,664	2 (0.00)	7 (0.01)	0 (0.00)	0 (0.00)
Only J	73,664	492 (0.67)	2,087 (2.83)	163 (0.22)	20 (0.03)
Only K	73,664	443 (0.60)	1,422 (1.93)	75 (0.10)	37 (0.05)
A + J	73,664	1 (0.00)	7 (0.01)	0 (0.00)	0 (0.00)
A + K	73,664	3 (0.00)	2 (0.00)	1 (0.00)	0 (0.00)
B + J	73,664	0 (0.00)	2 (0.00)	0 (0.00)	0 (0.00)
B + K	73,664	0 (0.00)	4 (0.01)	1 (0.00)	0 (0.00)
J + K	73,664	63 (0.09)	318 (0.43)	32 (0.04)	19 (0.03)
A + J + K	73,664	1 (0.00)	2 (0.00)	0 (0.00)	0 (0.00)
B + J + K	73,664	0 (0.00)	3 (0.00)	0 (0.00)	0 (0.00)

TSF, Taihe Silky Fowl; DGSC, Dongxiang Green-Shell Chicken; CPC, Chongren Partridge Chicken; NYC, Ningdu Yellow Chicken.

### 3.4 Phylogenetic analysis of ALV-K isolates from Jiangxi chickens

**Figure 2:**
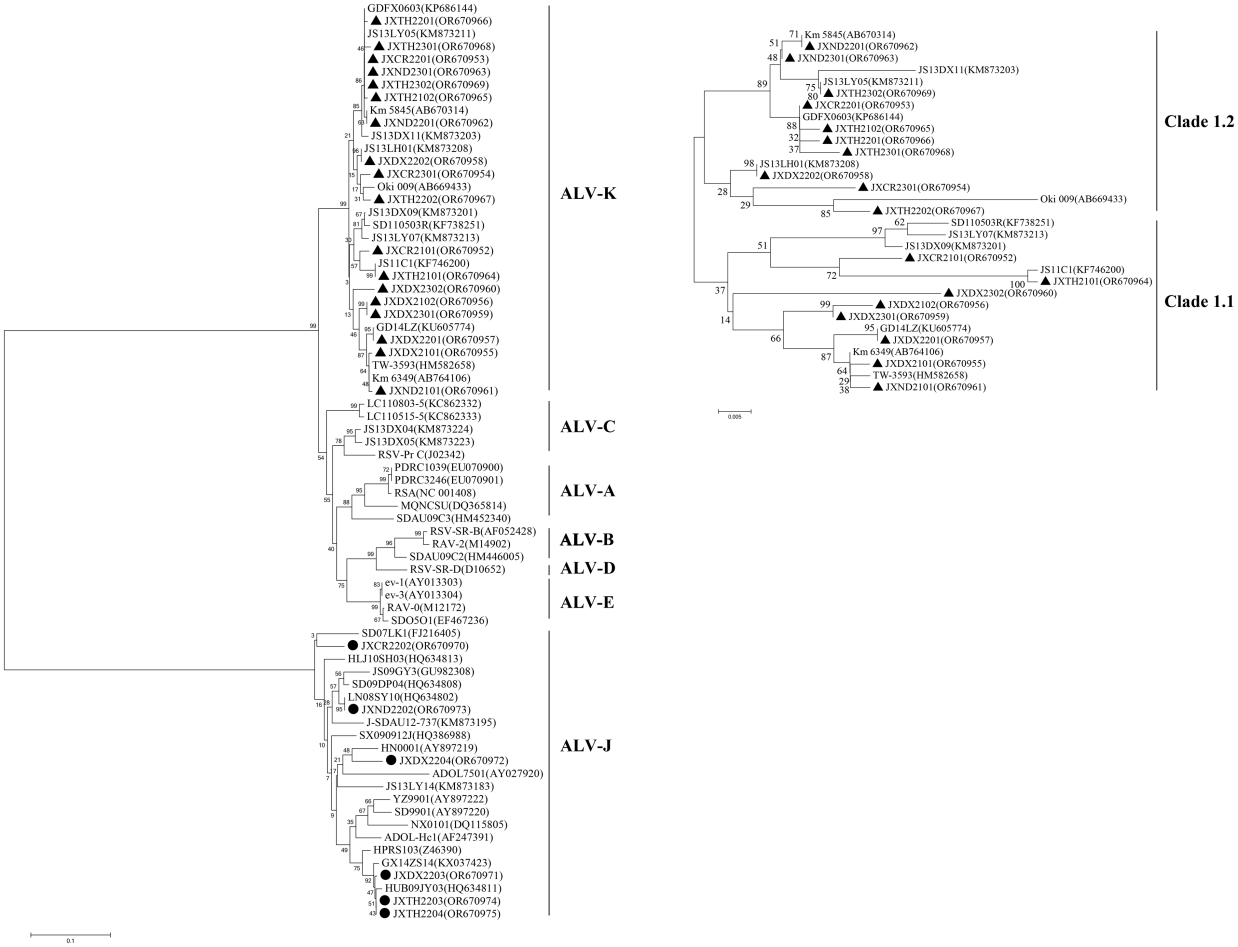
Phylogenic analysis of new isolates of ALV-K. Phylogenic analysis of the amino acid sequence encoded by the gp85 gene of ALV. Circles represent the new isolates. ALV-A, avian leukosis virus subgroup A; ALV-B, avian leukosis virus subgroup B; ALV-C, avian leukosis virus subgroup C; ALV-D, avian leukosis virus subgroup D; ALV-E, avian leukosis virus subgroup E; ALV-J, avian leukosis virus subgroup J; ALV-K, avian leukosis virus subgroup K.

During 2021–2023, 18 of the 2,436 ALV-K strains isolated were selected for whole genome sequencing. The results of the analysis showed that the overall nucleotide similarity of the gp85 gene of these 18 viruses ranged from 93.0 to 99.8% and amino acid similarity from 92.5 to 99.4%, while the nucleotide similarity with the other 13 strains of the K isoform ranged from 92.1 to 100% and amino acid similarity from 91.9 to 100%. The overall nucleotide similarities between the gp85 gene of the 18 viruses isolated in this study and those of ALV-A, ALV-B, ALV-C, ALV-D, ALV-E, and ALV-J were 83.1–87.6%, 82.2–84.0%, 86.5–91.5%, 82.5–84.1%, 79.8–87.6%, and 79.8–87.6%, and 41.2–44.2% amino acid similarities of 80.4–85.0%, 76.8–82.6%, 82.9–88.1%, 80.5–82.3%, 75.7–83.8%, and 33.9–38.2%, respectively (Table S2). According to the ALV subgroup analysis based on the gp85 gene sequence, all the above 18 isolates were clustered in one evolutionary branch, but were not in any cluster with the reference strains of subtypes A-E and were most distantly related to the reference strains of subgroup J, indicating that these 18 isolates belonged to ALV-K (Figure 2). Further analysis of all the above 18 isolates revealed that 10 ALV-K isolates were in Clade 1.2 and 8 ALV-K isolates were in Clade 1.1 (Figure 2).

### 3.5 Characteristics of gp85 of ALV-K isolates from Jiangxi local chicken

**Figure 3:**
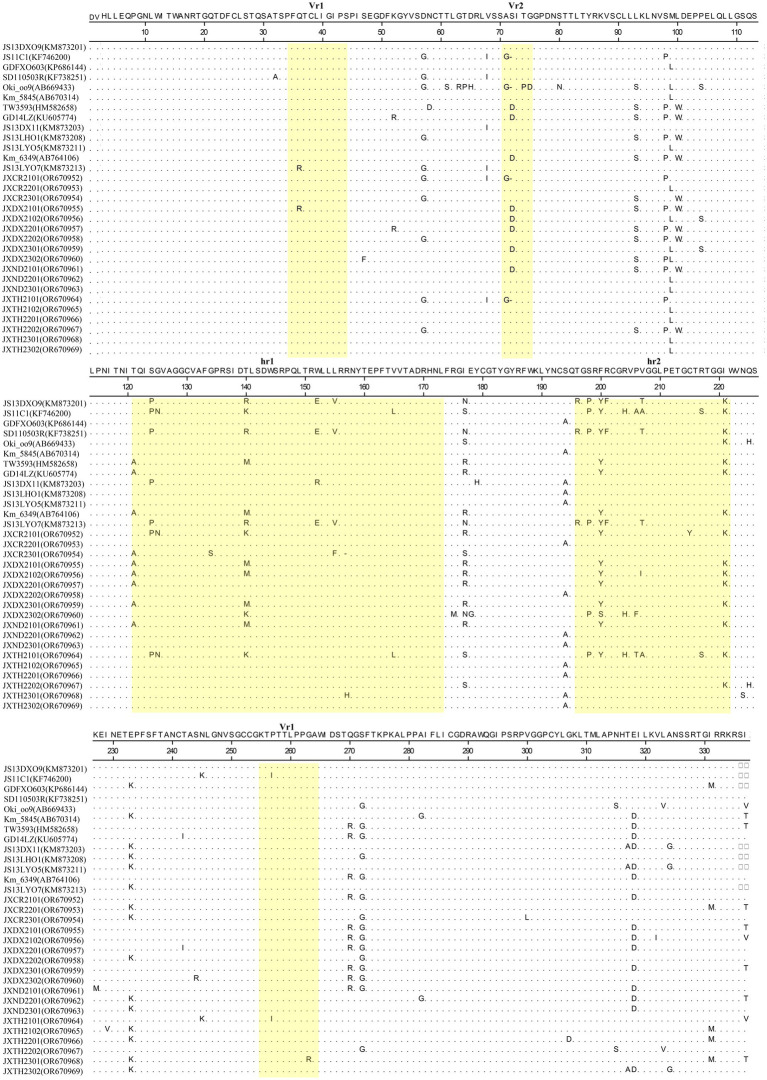
Mutations in the gp85 protein of ALV-K. Yellow squares represent hr1, hr2, Vr1, Vr2, and Vr3 of ALV-K. The dots indicate identical residues, the letters indicate substitutions, and the dashes indicate deletion. ALV-K, avian leukosis virus subgroup K.

The gp85 gene isolated in this study was 1,005 bp in length and encoded 335 amino acids, while the gp85 gene of JXCR2101, JXTH2101, JXCR2301 was 1,002 bp in length and encoded 334 amino acids. The amino acid differences between the strains were mainly concentrated in the two highly variable regions hr1 and hr2 and the variable region Vr2, including insertion, deletion and substitution mutations. Amino acid sequence comparison showed that JXCR2101 and JXTH2101 had a “D” deletion at position 72 compared to the other strains, and it is noteworthy that the JXCR2301 isolate in this study had a deletion from R at position 157. Importantly, the strains in this study showed different mutations from the reference strain, such as R mutation to H at position 155 of JXTH2302 at hr1, F mutation to S at position 200 of JXDX2302 at hr2, and P mutation to A at position 207 of JXDX2302 at hr2 (Figure 3).

### 3.6 Viral replication and competitive advantages of the isolates from Jiangxi local chicken

**Figure 4:**
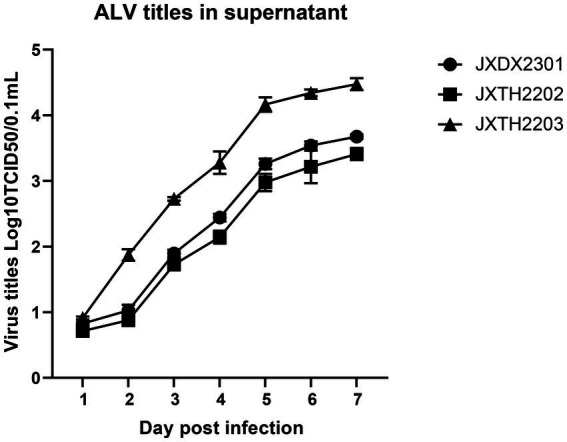
Comparison of proliferation and competitive advantages of different strains. Supernatants of different infected cells were harvested at 1–7 d after infection and then titered for 50% tissue culture infective dose (TCID50) using the Reed-Muench method.

Supernatants of the infected cells at different intervals were harvested and tested for viral titers using the Reed-Muench method. JXDX2301, JXTH2202, and JXTH2203 strains were inoculated with DF-1 cells, and the supernatants from infected cells at different time intervals were harvested and tested for viral titers using the IFA method, and viral virulence was calculated by the Reed-Muench method. As shown in Figure 4, from 1 to 7 dpi, the viral titer of JXTH2203 was higher than that of JXDX2301 and JXTH2202. the viral load in the chicken genome was detected using the single-copy housekeeping gene β-actin as a reference. At 3 dpi, JXDX2301, JXTH2202, and JXTH2203 had the highest significant viral titers (*p* < 0.0002), which were 1.844 and 2.042 times lower than the titer of JXTH2203, respectively.

## 4 Discussion

ALV-K infects chickens without clinical or only subclinical signs and has been considered non-pathogenic or low pathogenicity (Li Y. et al., 2022). At the same time, long-term targeted testing and genetic evolutionary analyses of isolates in the field are lacking. Since 2015, however, the finding of enhanced replication or increased pathogenicity of ALV-K isolates obtained from clinical samples has drawn the attention of researchers to changes in the genetic evolution and pathogenicity of ALV-K (Lv et al., 2019; Chen et al., 2022). Currently, ALV-K is prevalent not only in Chinese yellow chicken breeds, but also in chicken breeds from other Asian countries (Ochi et al., 2012; Chang et al., 2013; Guo et al., 2023). Compared with ALV-J, ALV-K is easily overlooked in routine testing, which is detrimental to the ALV eradication programmed (Chen X. et al., 2020). ALV-K, as the latest new subgroup to be discovered, has a strong transmission range and ability to spread even though its pathogenicity is weaker than that of the other subgroups, and it is especially more widespread in yellow-feathered broilers and local strains of chickens, which has resulted in some economic losses to the poultry industry (Li et al., 2019; Chen et al., 2022).

Until now, there is no report on the epidemiology of ALV-K in local chicken breeds in Jiangxi region. In this study, we reported for the first time the epidemiology of ALV and ALV-K gene characterization in local chickens in Jiangxi region during the period of 2021–2023, which can help us to understand the status of ALV-K in local chicken breeds in Jiangxi, and provide clues to the evolutionary trend of this virus. In this study, 35 strains of ALV-A, 13 strains of ALV-B, 3,200 strains of ALV-J, and 2,436 strains of ALV-K were isolated from 73,664 samples collected during the period of 2021–2023, which suggests that there are multiple subgroups of ALV infections in local breeds of chickens in the Jiangxi region at the present time, with ALV-J as the main subgroup. In recent years, epidemiological investigations of ALV disease have been carried out in many regions nationwide, and the results of serological studies showed that the infection rate of subpopulation A/B was between 5 and 10%, while the infection rate of subpopulation J was around 10–20% (Shao et al., 2017; Zhou et al., 2019; Dou et al., 2023). The results of the pathogenetic study showed that the infection rate of subgroup A/B ranged from 8.3–1.65% in local chickens, while the infection rate of subgroup J was 6.61% and that of subgroup K was 2.48% (Su et al., 2018b). In contrast, nucleic acid testing of some clinical samples with suspected disease revealed that ALV-J infection was significantly higher than the former. We found that the predominance of ALV-J and the co-infection of multiple subpopulations is the current epidemiological status of avian leukosis, which is basically consistent in different regions. It is worth noting that a new subpopulation, ALV-K, has emerged in recent years, and its prevalence in local chicken breeds is second only to that of ALV-J. These data suggest that ALV-J infections in local flocks are very common in China, a large poultry farming country. Although nationwide purification procedures for avian leukosis have been implemented for more than a decade, the disease has not been completely eradicated. Therefore, a continuous molecular epidemiological investigation of ALV infection in China is necessary.

From the 2,436 isolated ALV-K strains, 18 samples identified by PCR to be free of exogenous subgroups A, B and J ALV infections, and with only subgroup K ALV infections present, were randomly selected for whole-genome sequencing and phylogenetic analyses of the genomes. The results revealed that the 18 isolates isolated in this study were distributed on both major branches of ALV-K. Ten of these 27 isolates were concentrated in the Clade 1.2 branch of the ALV-K gp85 gene evolutionary tree, and eight were concentrated in the Clade 1.1 branch of the ALV-K gp85 gene evolutionary tree. Combining the isolation time and region of different strains, it can be learnt that the ALV-K strains in Jiangxi have genetic diversity (Guo et al., 2023). Among six isolates of silk-feathered silkie, five ALV-K isolates were in the Clade 1.2 branch, while among six isolates of Dongxiang green laying hen, five ALV-K isolates were in the Clade 1.1 branch. Interestingly, many current epidemiological investigations have concluded that the position of ALV isolates on the evolutionary tree is related to the genetic background of their hosts, i.e., ALVs of the same host origin tend to be more similar.

Sequence analysis of the coding region showed that the gag and pol genes remained highly conserved in all ALV-J strains. It was shown that the env gene and LTR sequences have an important influence on the replication rate and pathogenicity of ALV-K. Some of the amino acids encoded by the gp85 gene are also strongly associated with the cellular receptor for ALV-K. By analyzing the LTR sequences of the isolates, we found that the LTR sequences of the three isolates showed multiple deletions and mutations compared with those of the ALV-J strain, which has the fastest replication rate and the strongest pathogenicity, and ALV-K strains with exogenous ALV as the backbone and the env gene for ALV-K, such as JXCR2101, JXCR2201, and JXCR2301, resulting in a part of the deletions in CAAT LTR Enhancer Box, CAAT Box, PRE Box, CarG Box, and Y Box (Supplementary Figure S1). During initial virus isolation, the low replication efficiency of the isolates in this study may be related to the deletion of these transcription elements. Studies have shown that ALV-K strains such as JS15SG01 and HB2015032, which have exogenous ALV as the backbone and the env gene as ALV-K, are also more pathogenic (Liang et al., 2019; Li et al., 2021). In contrast, the isolate in this study, like most classical ALV-K strains in recent years, belongs to the category with endogenous ALV as the backbone and the env gene for ALV-K. ALV infection of host cells is dependent on the binding of its vesicle membrane surface protein, gp85, to the cellular receptor, and studies have shown that the ALV-K is identical to the ALV-A cellular receptor, Tva, and the ALV-K vesicle membrane protein (SU) hr2 region G at position 196 and R at position 198 determine whether ALV-K SU can bind to Tva (Prikryl et al., 2019; Chen J. et al., 2020). By analyzing the SU protein of the isolate in this study, it was found that the G and R at position 196 and 198 were conserved, and the Y/F at position 200 of the isolate JXDX2302 was mutated to S, which indicated that the structural domains of the receptor were conserved in the isolate in this study.

To confirm this speculation, strains JXDX2301, JXTH2202, and JXTH2203 were inoculated into DF-1 cells and the replication kinetics were recorded. It was found that the replication rate of JXTH2203 was significantly higher than that of JXDX2301 and JXTH2202, while JXTH2203 had a clear advantage in replication ability. Due to the low replication efficiency of JXTH2202, ALV-K is easily overlooked in the daily monitoring of chickens, leading to vertical transmission of the virus. This also reminds us that more measures should be taken to improve the detection sensitivity, especially when ALV clearance is performed on Chinese indigenous breeds.

## 5 Conclusion

To study the prevalence of each subtype of ALV in local chickens in Jiangxi, we tested 73,664 clinical samples from four major local chickens in Jiangxi from 2021 to 2023. The results showed that ALV-J was the most frequently detected virus with prevalence rates ranging from 1.47 to 8.49%, and ALV-K was the second most prevalent virus detected in blood samples. It is noteworthy that although ALV-K has only recently been detected, its prevalence is increasing every year and may pose a long-term threat to endemic flocks. We report for the first time the prevalence and genetic variation of ALV-K in local chicken breeds in Jiangxi, China. Phylogenetic analyses showed that in the past 3 years, local breeds of chickens in Jiangxi were genetically diverse in terms of the distribution of ALV-K in various branches of the evolutionary tree. ALV-K is easy to be ignored because of its low viral replication efficiency and difficult to isolate, and there is a risk of long-term cryptic transmission in chickens. Moreover, its current infection in local breeds of chickens in China is complex, and in-depth studies should be carried out on it, and it should not be ignored in the decontamination process to avoid it from becoming a potential risk.

## Data availability statement

The data presented in the study are deposited in the GenBank repository, accession number OR670952-OR670975.

## Ethics statement

The animal study was approved by Institute of Animal Husbandry and Veterinary Medicine, Jiangxi Academy of Agricultural Sciences. The study was conducted in accordance with the local legislation and institutional requirements.

## Author contributions

FZ: Data curation, Funding acquisition, Investigation, Writing – original draft, Writing – review & editing. HL: Methodology, Resources, Visualization, Writing – original draft. CL: Investigation, Methodology, Software, Supervision, Writing – original draft. YWe: Conceptualization, Formal analysis, Project administration, Supervision, Writing – original draft. WZ: Formal analysis, Resources, Visualization, Writing – original draft. YWu: Methodology, Project administration, Resources, Writing – original draft. ZK: Formal analysis, Funding acquisition, Methodology, Resources, Visualization, Writing – review & editing.
