# Supplementary material for: Detection and genetic diversity of subgroup K avian leukosis virus in local chicken breeds in Jiangxi from 2021 to 2023
Source: Front Microbiol. 2024 Feb 8;15:1341201. doi: 10.3389/fmicb.2024.1341201 (PMC10882074; doi:10.3389/fmicb.2024.1341201)
Supplement: Supplementary file 2 [file Table_1.docx]

Table S1 Reference sequences on ALV strains

| Strains | GenBank ID | Subtype | Origin | Year | Host |
| --- | --- | --- | --- | --- | --- |
| GX14NN02 | MNO66153 | J | GX | 2014 | YL |
| SDAU1704 | KY980660 | J | SD | 2017 | WB |
| K243 | KX6111833 | J | GD | 2016 | YL |
| GD1411-1 | KU500038 | J | GD | 2014 | YL |
| GD1407 | KU500035 | J | GD | 2014 | YL |
| GX14HG04 | KX058878 | J | GX | 2014 | YL |
| GDQY1201 | JX423792 | J | GD | 2012 | YL |
| SCAU11-XG | KC149971 | J | GD | 2012 | WB |
| GD1109 | JX254901 | J | GD | 2011 | WB |
| GX17YL05 | MNO66142 | J | GX | 2017 | YL |
| GX17NN05 | MNO66145 | J | GX | 2017 | YL |
| SCDY1 | HQ425636 | J | SC | 2010 | WB |
| SCGS-1 | JQ396302 | J | SC | 2010 | LC |
| HLJ13SH01 | KM376510 | J | HLJ | 2013 | WB |
| GD13HY | KU500031 | J | GD | 2013 | YL |
| GD15MM01 | MNO66154 | J | GX | 2015 | YL |
| GX15MM61 | MNO66150 | J | GX | 2015 | YL |
| GX16YL02 | MNO66147 | J | GX | 2016 | YL |
| JLO9H01 | HQ148554 | J | JL | 2009 | WB |
| YZ9902 | HM235670 | J | JS | 2009 | WB |
| HPRS103 | Z46390 | J | UK | 1988 | WB |
| WB11098 | JX848322 | J | HLJ | 2011 | WB |
| ADL-7501 | AYO27920 | J | USA | 2001 | WB |
| NM2002-1 | HM235670 | J | NMG | 2002 | Wb |
| MRL905 | JF951728 | J | Russia | 2008 | WB |
| ZB110604-6 | KC841157 | J | CHN | 2014 | BSF |
| LC110515-3 | KC841152 | J | CHN | 2014 | BSF |
| LC110515-4 | KC841153 | J | CHN | 2014 | BSF |
| RSA | NCO01408 | A | France | 1990 | - |
| SDAU09C1 | HM452339 | A | SD | 2010 | - |
| Schmidt-Ruppin B | AFO52428 | B | USA | 1998 | - |
| SDAU09C2 | JF826241 | B | SD | 2010 | - |
| Prague | J02342 | C | USA | 1977 | - |
| Schmidt-Ruppin D | D10652 | D | Japan | 1992 | - |
| Ev-1 | AY013303 | E | USA | 2000 | - |
| JS1208 | MT623675 | E | JS | 2020 | - |
| JS11C1 | KF746200 | K | JS | 2012 | - |
| JS13LY19 | MG770235 | K | JS | 2018 | - |
| TW-3593 | HM582658 | K | TW | 2013 |  |
| GD1701 | MK941182 | K | GD | 2017 |  |
| Oki_009 | AB669433 | K | Japan | 2007 |  |

GX: Guangxi, China; SD: Shandong, China; GD: Guangdong, China; SC: Sichuan, China ; JL: Jilin, China, JS: Jiangsu, China, HLJ: Heilongjiang. China; TW: Taiwan; CHN: China; USA: United States of America; UK: England; Japan: Japan; Russia: Russia. WB: White-Feather Broiler; Wb: wild bird; YL Yellow chicken; LC: Local chicken; BSF: Black bone silky fow
